# Pain sensitivity and tactile spatial acuity are altered in healthy musicians as in chronic pain patients

**DOI:** 10.3389/fnhum.2014.01016

**Published:** 2015-01-06

**Authors:** Anna M. Zamorano, Inmaculada Riquelme, Boris Kleber, Eckart Altenmüller, Samar M. Hatem, Pedro Montoya

**Affiliations:** ^1^Research Institute on Health Sciences, University of Balearic IslandsPalma de Mallorca, Spain; ^2^Department of Nursing and Physiotherapy, University of Balearic IslandsPalma de Mallorca, Spain; ^3^Institute of Medical Psychology and Behavioral Neurobiology, University of TübingenTübingen, Germany; ^4^Department of Music Physiology and Musician’s Medicine, University of Music, Drama and MediaHannover, Germany; ^5^Physical Medicine and Rehabilitation, Brugmann University HospitalBrussels, Belgium; ^6^Institute of Neuroscience, Université Catholique de LouvainBrussels, Belgium

**Keywords:** tactile threshold, pain sensitivity, chronic pain, musicians, somatosensory training

## Abstract

Extensive training of repetitive and highly skilled movements, as it occurs in professional classical musicians, may lead to changes in tactile sensitivity and corresponding cortical reorganization of somatosensory cortices. It is also known that professional musicians frequently experience musculoskeletal pain and pain-related symptoms during their careers. The present study aimed at understanding the complex interaction between chronic pain and music training with respect to somatosensory processing. For this purpose, tactile thresholds (mechanical detection, grating orientation, two-point discrimination) and subjective ratings to thermal and pressure pain stimuli were assessed in 17 professional musicians with chronic pain, 30 pain-free musicians, 20 non-musicians with chronic pain, and 18 pain-free non-musicians. We found that pain-free musicians displayed greater touch sensitivity (i.e., lower mechanical detection thresholds), lower tactile spatial acuity (i.e., higher grating orientation thresholds) and increased pain sensitivity to pressure and heat compared to pain-free non-musicians. Moreover, we also found that musicians and non-musicians with chronic pain presented lower tactile spatial acuity and increased pain sensitivity to pressure and heat compared to pain-free non-musicians. The significant increment of pain sensitivity together with decreased spatial discrimination in pain-free musicians and the similarity of results found in chronic pain patients, suggests that the extensive training of repetitive and highly skilled movements in classical musicians could be considered as a risk factor for developing chronic pain, probably due to use-dependent plastic changes elicited in somatosensory pathways.

## Introduction

Musical training involves the development of fine motor skills under high motivational drive linked to the emotional power of music (Koelsch et al., 2008; Jäncke, 2009). Acquisition of these specialized skills is achieved through highly repetitive and spatially stereotyped sensory-motor training over many years, which requires the simultaneous integration of multimodal sensory and motor information (Altenmüller, 2008). The prolonged and intensified processing of these sensory and motor inputs required for task performance induces modifications in the structural and functional organization of the somatosensory system (Münte et al., 2002; Kleber et al., 2010). Although these plastic brain changes are typically related to levels of expertize, it also has been reported that practice routine and extensive use may produce task-specific movement disorders such as focal dystonia (Altenmüller and Jabusch, 2010) and chronic pain (Steinmetz et al., 2014).

Indeed, according to several surveys, between 60% and 90% of professional musicians may experience musculoskeletal pain and pain-related symptoms during their careers as a consequence of playing an instrument (Middlestadt and Fishbein, 1988; Fry, 1989; Steinmetz et al., 2014), with an incidence of musculoskeletal pain that is even higher in music students (Brandfonbrener, 2009; Steinmetz et al., 2012). Overuse syndromes (or repetitive strain injuries) and inflammatory conditions are the most commonly diagnosed pain disorders in musicians (Fry, 1989; Steinmetz et al., 2014), and cervical pain is the most frequent symptom across all instrument groups (Paarup et al., 2011; Steinmetz et al., 2014). A variety of therapeutic and preventive approaches are frequently used to alleviate pain, including medication, physical therapy, exercises, stretching and long periods of absolute rest but unfortunately, recurrence rates after pain relief are still high (Fry, 1988; Shafer-Crane, 2006). In summary, these clinical and epidemiological data suggest that the extensive and repetitive sensory-motor training in professional musicians may present a precursor for development and chronification of pain symptoms in this highly skilled group.

During the last decades, a number of studies have shown interest for psychophysiological and psychophysical correlates of pain sensitivity and function of the somatosensory system in different pain disorders (Maier et al., 2010; Backonja et al., 2013). Enhanced sensitivity to painful stimuli has been suggested as a characteristic feature of pain syndromes, such as chronic back pain (Puta et al., 2013). Moreover, impaired tactile acuity has been highly associated to chronic pain conditions (Flor et al., 1997; Moriwaki and Yuge, 1999; Juottonen et al., 2002; Johnston et al., 2008; Moseley, 2008). In musicians, pain threshold assessments in pianist, violist and violinist players with neck pain have shown thermal and mechanical hyperalgesia not only over the painful areas but also over non-symptomatic areas distant to the painful region (Linari-Melfi et al., 2011; Steinmetz and Jull, 2013). These findings suggest that an extensive sensorimotor training, such as musical training, can lead to increase pain sensitivity, probably as a consequence of somatosensory cortical reorganization. However, psychophysiological and psychophysical evaluations in professional musicians are still scarce and no study has yet investigated the nociceptive pain response together with tactile sensitivity evaluations in professional musicians. Furthermore, previous studies have hinted towards a strong psychosomatic influence. It has been reported that musicians with chronic pain premorbidly had higher free floating anxiety scores and panic attacks than pain free musicians (Jabusch et al., 2004). The current work is an exploratory study aiming to examine the complex interactions between musical training and chronic pain, by assessing tactile thresholds and pain sensitivity to pressure and thermal stimuli with standardized observation methods in four groups of subjects: professional classical musicians with and without chronic pain and non-musicians with and without chronic pain. It was hypothesized that the effects of extensive musical training could predispose professional musicians to display an altered perception of painful and non-painful somatosensory stimuli similar to that of chronic pain patients. We additionally hypothesized that psychological factors, such as proneness to anxiety could have an impact on pain sensitivity.

## Materials and methods

### Participants

Seventeen professional classical musicians with chronic back pain (seven women, 30 ± 9.4 yrs) and 30 pain-free professional classical musicians (ten women, 27 ± 8.9 yrs) were recruited from different music schools and orchestras in the Balearic Islands (Spain). All musicians participating in the study were conservatory trained orchestra instrumentalists (string, brass or wind instruments), with long musical professional practice. The type of instrument played, daily practice duration, and life-time practice are described in Table 1. In addition, 20 non-musicians with chronic back pain (eight women, 32 ± 8.7 yrs) and 18 pain-free non-musicians (seven women, 28 ± 5.1 yrs) were recruited from the University of the Balearic Islands. Exclusion criteria for all groups were: presence of a history of trauma or neurologic entrapment syndromes to the arm regions, neurological disease or pregnancy. To better explore central sensitization, we aimed at assessing tactile and pain sensitivity over a non-symptomatic area distant to the participant’s painful region. For this reason, subjects with peripheral pain conditions of upper limbs were discarded. This exclusion criterion has been used previously in studies assessing pain sensitivity (Puta et al., 2013; Steinmetz and Jull, 2013).

**Table 1:** **Professional characteristics of musicians according with the presence of chronic pain: type of instrument, years of practice and average hours of daily practice**.

Musicians	Chronic pain (*n* = 17)	Pain-free (*n* = 30)
Keyboard (*n*)	0	1
Strings (*n*)	7	6
Plucking instruments(*n*)	6	7
Woodwinds instruments (*n*)	4	4
Brass instruments (*n*)	0	12
Years of practice (mean ± SD)	21 ± 8.0	20 ± 8.7
Average hours of daily practice (mean ± SD)	4.5 ± 2.2	4.3 ± 1.1

Subjects with chronic back pain included into the study fulfilled the following criteria: (1) more than 6-months history of persisting back pain; and (2) absence of structural abnormalities within the lumbar spine. At the time of recruitment, all participants were verbally informed about the details of the study and provided written consent. The study was performed in accordance with the Declaration of Helsinki (1991) and approved by the Ethics Committee of the Balearic Islands.

### Assessment of clinical characteristics of pain

Participants completed the Beck’s Depression Inventory II (Beck et al., 1961) and the State-Trait Anxiety Inventory (Spielberger et al., 1970) for assessing mood state and proneness to anxiety and the Edinburgh Handedness Inventory for manual dominance (Oldfield, 1971). In addition, all participants with chronic pain underwent a semi-structured clinical interview, including questions about duration and pain intensity, location, and psychosocial factors involved in the maintenance of pain (West-Haven Yale Multidimensional Pain Inventory of Pain—WHYMPI, (Kerns et al., 1985), as well as questions about musical practice (years of playing, daily practice hours, age of onset of music training).

### Measurement of tactile thresholds and pain sensitivity

#### Tactile thresholds

Tactile thresholds were bilaterally assessed in all participants at the bottom of the middle finger pad with the aim of avoiding the callosity in the musicians’ finger-tips. The tasks were always completed in the following order: (1) mechanical detection; and (2) tactile spatial acuity; and (3) two-point discrimination. The presentation sequence for stimulation side (right vs. left hand) was individually determined at the beginning of the session by chance (throwing a coin in the air).

Mechanical detection thresholds were measured with a kit of von Frey monofilaments (Somedic Sales AB, Hörby Sweden) consisting of 17 nylon hairs with increasing diameters ranging in tactile pressure-equivalent from 0.5 to 1078 mNewtons (mN). They were applied by touching the skin in a perpendicular way, pressing it slowly down until it buckles, holding it steady during 1.5 s and removing it in the same way as it was applied. After several trials to assure the understanding of the procedure, subjects were instructed to close their eyes during the procedure and answer “yes” when a touch stimulus was perceived. Threshold score was calculated according to the method of limits (Backonja et al., 2013). The procedure started with the thickest filament in descending order and stopped when the subject perceived the lowest pressure. To control for attentional effects on localization accuracy and false positive responses, additional null stimuli were performed in other non-target areas, including the finger pad of index and ring fingers. The final threshold was calculated as the mean of the two thinnest filaments positively detected by the subject within 3 s of the stimulus. Trials with a response delay greater than 3 s were considered invalid and repeated. Null stimuli were not taken into account for the calculation of the final threshold. Thresholds were log10-transformed before statistical analyses. This procedure has previously been used to assess tactile thresholds in healthy subjects and patients with chronic pain (Martínez-Jauand et al., 2013; Puta et al., 2013) and individuals with cerebral palsy (Riquelme et al., 2013).

For measurement of grating orientation thresholds, an extended set of 11 hemispherical JVP domes (Van Boven and Johnson, 1994) consisting of grating surfaces with equidistant widths of bars and grooves (0.35, 0.5, 0.75, 1, 1.2, 1.5, 2 and 3 mm) (Stoelting Inc., Wood Dale, IL, USA) were used. Domes were placed during 2 s provoking a skin deformation of about 2 mm (Van Boven and Johnson, 1994; Bara-Jimenez et al., 2000). Subjects were required to identify the orientation of the grooves (along or across the longitudinal axis of the finger pad) after removal of the dome and asked to indicate how the grooves were oriented. Each orientation was presented 20 times (two series of 10 times) in pseudo-randomized order. To avoid order effects and habituation, the two series of the spatial discriminations task were intercalated with the two series of the two point discrimination task described below. Testing proceeded from the widest grating dome (3 mm) to the next thinnest one until the performance level dropped below 75% correct discrimination. Grating orientation thresholds were computed as a simple linear interpolation estimate of the 75% correct grating width with the following formula:

$$\text{threshold}=w^{-}+\left(w^{+}-w^{-}\right)*\left(0.75-p^{-}\right)/\left(p^{+}-p^{-}\right)$$

where *w*^−^ and *w*^+^ were the largest width that achieved less than 75% of correct answers, and the smallest width that achieved more than 75% of correct answers, respectively. The *p*^−^ and *p*^+^ values were the fraction of correct responses at *w*^−^ and *w*^+^, respectively. Subjects were assigned a threshold value of 3 mm when they were unable to achieve 75% of correct responses on the widest grating dome (Sanger et al., 2001). Grating orientation thresholds have been used previously to measure tactile accuracy in healthy subjects and patients who perform high repetitive movements (Bara-Jimenez et al., 2000; Sanger et al., 2001).

Two-point discrimination thresholds were measured by using seven pairs of needles (diameter 200 microns) with fixed separation distances of 1, 2, 3, 4, 5, 6 and 7 mm (Discriminator, Sammons Preston, USA). A single needle probe was used as control. The needles were mounted on a rotatable disk that allowed switching rapidly between distances. The procedure was applied in ascending order of distances and repeated twice. The subjects were instructed to close their eyes during the procedure and answer immediately if a sensation of one or two pricks was felt by saying “one” or “two”. The test started with the distance of 1 mm between the two needles, and then the distance between them was progressively increased until the participant was able to perceive two points instead of one. Null non-touch and non-change stimuli trials were used to ensure that participants were not guessing. According to the methods of limits, the lowest distance between those points in which participant reported feeling two stimuli on two consecutive trials was considered as the two-point discrimination threshold. This procedure has been used previously to assess tactile accuracy in healthy subjects and patients with chronic pain (Moseley et al., 2008).

#### Pain sensitivity

Pain sensitivity was bilaterally assessed at the bottom of the index finger pad (avoiding the callus) by using three experimental tasks: heat pain, pressure pain, and cold pain. The pressure pain task was always preceded and followed by the thermal pain task (heat or cold pain). In addition, half of participants began with the heat pain task, and the other half with the cold pain task. The presentation sequence for stimulation side (right vs. left hand) was individually determined at the beginning of the session by chance (throwing a coin in the air). For all three tasks, the method of limits was applied. After each measurement, participants were instructed to rate their pain intensity at threshold by using a 0–100 numerical rating scale (NRS) with 0 representing “no pain” and 100 “worst pain imaginable”, in order to assess the personal perception of pain. The ratio between subjective pain rating elicited by stimuli and stimulus intensity was computed as sensitivity score. A similar procedure has been previously described for assessing pain sensitivity in healthy subjects and patients with chronic pain (Martínez-Jauand et al., 2013; Puta et al., 2013).

Pressure pain sensitivity was measured with a digital dynamometer using a flat rubber tip (1 cm^2^; Force One, Wagner Instruments, Greenwich, CT USA). The force was applied by touching the skin in a perpendicular way. The test started with the contact of the device with the skin, and then the force increased until the participant perceived the stimulus as painful (pain threshold). Then, subjects reported their subjective pain rating. The pressure pain sensitivity score was defined as the ratio between subjective pain rating elicited by stimuli and amount of pressure in Newtons (N). The maximal force allowed was 140 N.

Heat pain sensitivity was measured with a computer-controlled contact thermal stimulator (Cold/warm plate AHP-301CPV, Teca, Schubert, IL, USA). Participants were instructed to keep the finger pad in contact with the thermal plate and to retract it as soon as the stimulation became painful. Temperature increased from a non-painful lukewarm temperature of 37°C at a mean rate of 0.2°C/s up to a maximum temperature of 52°C. The thermal pain sensitivity score was defined as the ratio between the perceived pain intensity (NRS) and temperature (°C) at which subjects first perceived heat pain (heat threshold).

For the measurement of cold pain sensitivity, the thermal plate was set at a constant temperature of 0°C. Participants were instructed to keep the finger pad in contact with the thermal plate base and to retract it when the cold sensation first became painful (maximal testing time: 180 s). The cold pain sensitivity score was defined as the ratio between subjective rating of pain intensity (NRS) and time (s) when subjects first perceived cold pain.

Tactile thresholds and pain sensitivity scores were always performed by the same researcher (AZ) in a quiet room with stable room temperature, and avoiding the application of stimuli on the callus of musicians’ fingertips. Pain sensitivity tasks were always preceded by the measurement of tactile thresholds.

### Statistical analysis

Analyses of variance (ANOVAs) were used to assess the effects of between subjects-factors MUSICIAN (musicians vs. non-musicians) and PAIN (chronic pain vs. pain-free individuals), and within-subjects factors HEMIBODY (left vs. right hand) on tactile and pain thresholds. In addition, ANOVAs were used to test the effects of between subjects-factors MUSICIAN (musicians vs. non-musicians) and PAIN (pain vs. pain-free individuals) on age, psychological (STAI and BDI questionnaires) and pain-related (WHYMPI) variables, as well as on data about musical practice. Significant interaction effects were further examined by using *post hoc* mean pairwise comparisons (Bonferrroni). For all ANOVAs, statistic indexes were corrected using Levene tests and Greenhouse–Geisser epsilons to account for violations of sphericity and homocedasticity assumption. Chi-square tests were used for testing the distribution of males and females on the groups. Pearson bilateral correlations were use to explore correlations between tactile and pain variables. The statistical significant level was set at *p* < 0.05. All statistical analyses were carried out with SPSS version 19 (SPSS Inc., Chicago, IL, USA).

## Results

Chi-square tests revealed that the distribution of males and females was similar in any of the subgroups formed by the combination of MUSICIAN and PAIN factors. Moreover, ANOVAs revealed no significant effects due to MUSICIAN, PAIN or MUSICIAN × PAIN on age.

Significant effects due to PAIN were found on depression (*F*_(1,77)_ = 9.56, *p* < 0.01), state- (*F*_(1,78)_ = 9.27, *p* < 0.01) and trait-anxiety (*F*_(1,77)_ = 8.19, *p* < 0.01). No significant effects due to MUSICIAN or MUSICIAN × PAIN were observed on these variables. Pairwise comparisons were done in order to explore if these differences due to the presence of chronic pain were found between musicians with and without chronic pain as reported in previous papers (Jabusch et al., 2004). *Post hoc* mean comparisons indicated a trend that anxiety (state and trait) was higher in musicians with chronic pain (state: *p* < 0.01; trait: *p* < 0.01) than in pain free-musicians, and that there were no differences between the two subgroups of non-musicians (pain-free and individuals with chronic pain). Moreover, patients non-musicians with chronic pain displayed higher depression (*p* < 0.01) scores than pain-free individuals (Table 2).

**Table 2:** **Socio-demographic and clinical characteristics of musicians and non-musicians according with the presence of chronic pain**.

	Musicians		Non-musicians	
	Chronic pain (*n* = 17)	Pain-free (*n* = 30)	Chronic pain (*n* = 18)	Pain-free (*n* = 20)
Age (y)	30.2 ± 9.4	27.5 ± 8.9	32.8 ± 8.7	28.2 ± 5.1
Gender (F/M)	7/10	10/20	8/10	7/10
Dominant hand (L/R)	3/14	1/29	1/17	2/18
Pain rating (0–10)	3.5 ± 1.6	N/A	3.2 ± 2.1	NA
Duration of pain (years)	10.3 ± 9.5	N/A	8.1 ± 4.6	NA
Depression	9.3 ± 5.9	6.3 ± 4.9	10.9 ± 9.5	4.6 ± 5.2
State anxiety	18.6 ± 11.7	10.1 ± 6.0	14.0 ± 9.2	10.9 ± 7.7
Trait anxiety	23.1 ± 9.6	15.2 ± 8.5	20.7 ± 9.9	16.4 ± 9.2
**WHYMPI**
Social support	3.0 ± 2.0	N/A	3.0 ± 1.4	N/A
Affective distress	0.9 ± 1.2	N/A	1.5 ± 1.2	N/A
Interference social activities	0.9 ± 1.1	N/A	1.5 ± 1.3	N/A
Interference daily activities	2.5 ± 1.0	N/A	2.5 ± 1.1	N/A
Pain intensity	2.0 ± 1.2	N/A	2.5 ± 0.9	N/A
Life control	3.4 ± 1.4	N/A	3.9 ± 1.0	N/A
Distracting responses	2.8 ± 1.2	N/A	2.4 ± 1.0	N/A
Solicitous responses	1.8 ± 1.2	N/A	1.9 ± 0.9	N/A
Punishing responses	0.9 ± 1.1	N/A	0.3 ± 0.4	N/A
Household chores	2.9 ± 1.2	N/A	3.1 ± 0.7	N/A
Activity away from home	2.4 ± 1.2	N/A	2.7 ± 0.7	N/A
Outdoor work	1.9 ± 1.7	N/A	1.9 ± 1.0	N/A
Social activities	2.1 ± 1.1	N/A	2.7 ± 0.7	N/A

*Values are mean ± SD or as otherwise indicated*.

*Abbreviations: F, female; M, male; L, left; R, right; N/A, not applicable*.

For musicians, ANOVAs with the between-subjects factor PAIN revealed that there were no differences between pain-free musicians and musicians with chronic pain on years of playing, daily practice hours, age of onset of music training. For individuals experiencing chronic pain, ANOVAs with the between-subjects factor MUSICIAN revealed that there were no differences between musicians and non-musicians with chronic pain on pain intensity ratings, pain duration and pain interference (WHYMPI).

### Tactile thresholds

**Figure 1:**
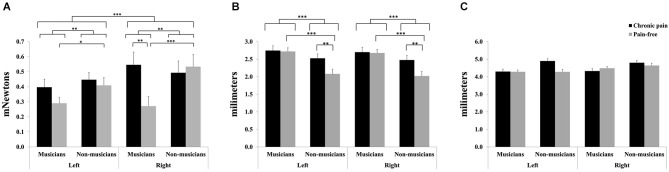
**Tactile sensitivity parameters in professional musicians and non-musicians, both with chronic back pain and pain-free**. Pain-free musicians showed the greatest touch sensitivity **(A)**, together with reduced spatial discrimination acuity **(B)**. No significant differences were found on two-point discrimination between groups **(C)**. Significant differences between groups are marked with asterisks (*p < 0.05, **p < 0.01, ***p < 0.001).

For mechanical detection thresholds, significant main effects due to HEMIBODY (*F*_(1,81)_ = 12.28, *p* < 0.001) and MUSICIAN (*F*_(1,81)_ = 9.25, *p* < 0.01) revealed that tactile sensitivity was overall higher (lower thresholds) at left than at right hand, and higher in musicians than in non-musicians. Furthermore, a significant HEMIBODY × MUSICIAN × PAIN (*F*_(1,81)_ = 6.34, *p* < 0.05) was yielded. *Post hoc* mean comparisons indicated that tactile sensitivity to mechanical stimuli was higher (i.e., lower mechanical thresholds) in pain-free musicians than in musicians with chronic pain at the right hand (*p* < 0.01), and that there were no differences between the two subgroups of non-musicians (pain-free and individuals with chronic pain), or at the left hand. Moreover, *post hoc* tests revealed that tactile sensitivity was higher (i.e., lower mechanical thresholds) in musicians than in non-musicians, indicating also that the effects appeared bilaterally in the subgroup of pain-free individuals (right hand: *p* < 0.001; left hand: *p* < 0.05), but not in the subgroup of chronic pain patients (Figure 1A).

For grating orientation thresholds, significant effects of MUSICIAN (*F*_(1,81)_ = 17.0, *p* < 0.001), PAIN (*F*_(1,81)_ = 5.1, *p* < 0.05), and MUSICIAN × PAIN (*F*_(1,81)_ = 4.11, *p* < 0.05) were yielded (Figure 1B). *Post hoc* mean comparisons revealed that pain-free individuals displayed higher spatial discrimination acuity (i.e., lower grating orientation thresholds) than chronic pain patients within non-musicians (*p* < 0.01), but not within musicians. Moreover, non-musicians had higher spatial discrimination acuity (i.e., lower grating orientation thresholds) than musicians within pain-free individuals (*p* < 0.001), but not within chronic pain patients.

No significant effects were yielded on two-point discrimination thresholds (Figure 1C).

### Pain sensitivity

**Figure 2:**
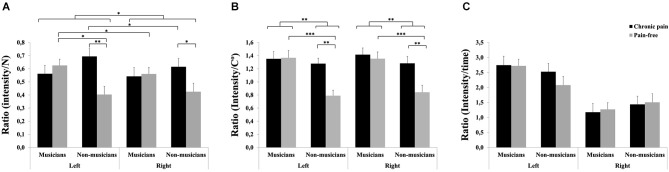
**Pain sensitivity to different stimuli in professional musicians and non-musicians, both with chronic back pain and pain-free**. Pain-free musicians showed the greatest pain sensitivity responsiveness together with chronic pain patients (musicians and non-musicians) at the different modalities: Pressure pain stimuli **(A)** and heat pain stimuli **(B)**. No significant differences were found on cold pain stimuli between groups **(C)**. Significant differences between groups are marked with asterisks (*p < 0.05, **p < 0.01, ***p < 0.001).

For pressure pain, significant effects due to HEMIBODY (*F*_(1,81)_ = 4.17, *p* < 0.05), MUSICIAN × PAIN (*F*_(1,81)_ = 5.92, *p* < 0.05) and HEMIBODY × MUSICIAN × PAIN (*F*_(1,81)_ = 4.25, *p* < 0.05) were found (Figure 2A). *Post hoc* pairwise mean comparisons of the interaction effects revealed that non-musicians with chronic pain displayed higher pressure pain sensitivity scores than pain-free non-musicians at both hands (*p* < 0.05), whereas there were no differences between the two subgroups of musicians on pressure pain sensitivity. Moreover, pain-free musicians had higher pressure pain sensitivity scores than pain-free non-musicians at the left hand (*p* < 0.05), whereas there were no differences at the right hand, or between musicians and non-musicians with chronic pain. Finally, the left hand was more sensitive to pain than the right hand in pain-free musicians (*p* < 0.05) and non-musicians with chronic pain (*p* < 0.05), whereas there were no hemibody differences within the other two subgroups.

For heat pain, significant effects due to MUSICIAN (*F*_(1,81)_ = 10.64, *p* < 0.01), PAIN (*F*_(1,81)_ = 6.06, *p* < 0.05) and MUSICIAN × PAIN (*F*_(1,81)_ = 4.94, *p* < 0.05) also revealed that chronic pain patients displayed higher pain sensitivity scores than pain-free individuals within non-musicians (*p* < 0.01), but not within musicians (Figure 2B). Moreover, musicians had higher sensitivity scores than non-musicians within pain-free individuals (*p* < 0.001), but not within chronic pain patients.

No significant effects were yielded on sensitivity scores to cold pain (Figure 2C).

In addition, we found a general positive correlation between tactile spatial acuity thresholds and heat pain ratios in right and left side (*r* = 0.29 and *r* = 0.28 respectively; *p* < 0.01), i.e., lower tactile acuity could be associated with increased sensitivity to pain. Moreover, significant correlations were found between two-point discrimination thresholds in right hand and pain pressure ratio in left hand in musicians with chronic pain (*r* = −0.53, *p* < 0.05) and pain-free musicians (*r* = −0.36, *p* < 0.05); whereas non-musicians with chronic pain showed significant correlations between two-point discrimination in left hand and pain cold ratio in left hand (*r* = −0.45, *p* < 0.05) and two-point discrimination in left hand and pain cold ratio in right hand (*r* = 0.49, *p* < 0.05). No correlations between tactile and pain variables were found for pain-free non-musicians.

## Discussion

The present study aimed at exploring the complex interactions between musical training and chronic pain, testing if altered somatosensory response associated with musical practice could mirror tactile and pain sensitization typical of chronic pain patients. For this purpose, tactile (mechanical detection, grating orientation, and two-point discrimination thresholds) and pain sensitivity tasks (subjective ratings to pressure, heat, and cold stimuli) were assessed in musicians with chronic back pain, pain-free musicians, non-musicians with chronic back pain, and pain-free non-musicians. Our results showed that pain-free musicians compared to non-musicians displayed a significant enhancement of tactile sensitivity (i.e., low detection thresholds), together with reduced spatial discrimination acuity (i.e., high grating orientation thresholds) and enhanced sensitivity to pressure and heat pain. Also, we found that these data were similar to results found in chronic pain patients groups (musicians and non-musicians), who also showed a significant reduction of tactile spatial sensitivity and increased sensitivity to pressure and heat pain stimulus compared to non-pain groups. In addition, our results also indicated that the effects of chronic pain and music training on mechanical tactile sensitivity were more prominent in the right hand, whereas group differences on sensitivity to pressure pain were lateralized to the left hand. In summary, our results revealed a global reduction in tactile sensitivity and simultaneous increase of pressure and thermal sensitivity in classic musicians, which could indicate that extensive sensorimotor training could potentially lead to somatosensory reorganization predisposing musicians to develop increased pain perception.

### Differential effects of music training on tactile and pain sensitivity in chronic pain patients and pain-free individuals

The acquisition of expert music performance requires extensive training over many years, typically starting during early infancy and continuing throughout life with stages of increasing physical and technical complexity (Altenmüller, 2008). This accumulates to up to 10.000 h of deliberate practice by the age of 20 (Ericsson et al., 1993) for developing excellent fine-motor control over pressure and force on keys, strings, bow or pistons (Askenfelt and Jansson, 1992), which leads to increased tactile sensitivity (Ragert et al., 2004). At the neural level, previous research has shown that sensorimotor learning and skill acquisition in musicians results in enlarged cortical receptive fields and altered sensorimotor mappings reflecting those parts of the body that are most frequently used (Elbert et al., 1995; Schwenkreis et al., 2007). Moreover, musicians’ cortical responses to somatosensory stimulation are enhanced in the presence of musical feedback from the instrument of training, showing strong cross-modal plasticity (Schulz et al., 2003). However, repetitive sensorimotor training can also provoke maladaptive neuroplasticity. Primate studies have provided strong evidence that long-lasting repetitive movements of the hand can also lead to degradation and dedifferentiation of receptive fields (Byl et al., 1996; Buonomano and Merzenich, 1998). Such effects have been extensively studied in musicians with focal dystonia (Bara-Jimenez et al., 2000; Altenmüller and Jabusch, 2010).

In the present study, significant differences between musicians and non-musicians were yielded on tactile thresholds and pain sensitivity measures in pain-free individuals, but not in individuals with chronic pain. The observed enhancement of tactile mechanical sensitivity (i.e., reduction of mechanical detection thresholds) found in pain-free musicians compared to pain-free non-musicians is in line with previous reports on experience dependent neuroplasticity (Elbert et al., 1995; Ragert et al., 2004). Musicians (pain and pain-free) also showed higher pain sensitivity that was associated with hightened tactile sensitivity even in distant body locations. It could be possible that the compelling similarities in spatial acuity and pain sensitivity measures found between musicians (pain vs. pain-free) represent a confusion of somatosensory inputs produced by expanded somatosensory receptive fields as a consequence of music training. Alternatively, the abnormal enhancement of tactile thresholds and sensitivity to heat and pressure pain in both musicians with and without chronic pain could also suggest that chronic pain and music training may exert similar effects on the processing of bodily information due to common plastic changes in somatosensory pathways. On the other hand, the fact that pain-free musicians displayed a significant enhancement of sensitivity to pain stimuli may corroborate the idea that professional musicians are exposed to a higher risk for developing chronic pain due to their extensive training of repetitive and highly skilled movements as suggested by clinical observations and several surveys (Middlestadt and Fishbein, 1988; Fry, 1989; Brandfonbrener, 2009; Steinmetz et al., 2014). This would be in contrast with studies showing a reduction of pain sensitivity after sensoriomotor training (Moseley et al., 2008; Riquelme et al., 2013). The high intensity of musical training and its consequent maladaptative plasticity reported by previous studies (Bara-Jimenez et al., 2000; Altenmüller and Jabusch, 2010) may be the causes for this apparent contradiction. Furthermore, selective attention and hypervigilance to sensory stimuli are enhanced in both chronic pain patients (Dehghani et al., 2003) and musicians (Dayan and Cohen, 2011; Strait and Kraus, 2011), which may contribute to the facilitation of pain perception in otherwise pain-free highly skilled instrumentalists.

Although tactile mechanical sensitivity (as measured by tactile detection thresholds to mechanical stimuli) was enhanced as consequence of music training, we also observed that tactile spatial discrimination acuity (as measured by grating orientation thresholds) was significantly reduced in musicians as compared with non-musicians. Such discordant results could be related to the activation of different somatosensory pathways. Thus, tactile punctate stimuli elicited by monofilaments seem to activate large myelinated Aβ sensory fibers (Courtney et al., 2010), whereas grating orientation patterns are independent of the contact force over the skin and could preferentially stimulate peripheral slow-adapting type 1 afferents (Johnson, 2001). Moreover, abnormal grating orientation acuity has been found in subjects with enlarged tactile receptive fields due to repetitive movements (e.g., patients with writer’s dystonia), suggesting that reduced spatial resolution could be a specific indicator of cortical reorganization (Bara-Jimenez et al., 2000; Sanger et al., 2001). Our results further suggest that extensive repetitive sensorimotor training over years might induce relevant dysfunctional changes in the processing of painful and non-painful bodily information, as it occurs with chronic pain.

### Differential effects of chronic pain on tactile and pain sensitivity in musicians and non-musicians

Previous studies have repeatedly shown that chronic pain is significantly associated with reduced ability to identify temporal and spatial characteristics of tactile stimuli (Flor et al., 1997; Moriwaki and Yuge, 1999; Juottonen et al., 2002; Johnston et al., 2008; Moseley, 2008). Moreover, it has been demonstrated that heightened pain sensitivity is a characteristic feature of several pain syndromes, such as chronic back pain (Puta et al., 2013), fibromyalgia (Martínez-Jauand et al., 2013) or phantom limb pain (Flor et al., 1995). In this sense, our results in non-musicians with chronic pain are fully in agreement with previous findings and might be interpreted as result of a generalized central sensitization elicited by persistence of pain over time. More interestingly, however, is the fact that musicians with chronic pain displayed less tactile sensitivity to mechanical stimuli (i.e., higher tactile mechanical detection thresholds) than pain-free musicians as this suggests that chronic pain might induce additional long-lasting effects in the processing of bodily information to those already elicited by music training in professional musicians.

With respect to psychometric assessments, chronic pain musicians showed higher depression and state and trait-anxiety values than pain-free individuals. This finding is consistent with the association between anxiety and chronic pain syndromes in musicians described in previous reports (Jabusch et al., 2004). Jabusch and colleagues demonstrated that musicians with chronic pain have increased free floating anxiety compared to healthy musicians. Furthermore, in this study they tried to retrospectively assess psychological states and could convincingly demonstrate that this condition had been prior to the commencement of the pain syndrome. In this sense, our results indicate that anxiety in musicians could act as a triggering factor for developing pain symptoms.

To our knowledge, this is the first study showing that repetitive and skilled movements may lead to changes in tactile sensitivity together with subjective responses to pain stimuli in pain and pain-free professional musicians. However, this study has several limitations that should be taken into account for the interpretation of the results. It should be noted that our findings were obtained on male and female adults, and due to the small subsamples no information about the modulatory effect of gender on the association between music training and chronic pain could be explored. Similarly, the subgroups of professional classic musicians included in the study were small and composed of experienced players of string, plucking, keyboard, brass, and woodwind instruments, which demand different levels of skilled movements of the hands. Although there were no significant differences in the distribution of chronic pain among these musicians, we cannot rule out the possibility that chronic pain was differentially affecting tactile thresholds and pain sensitivity depending on the type of instrument played. Our findings of lateralized effects on tactile thresholds and pain sensitivity could be also due to these differences and were therefore not further discussed. Moreover, our study population was only comprised of classical musicians with a formal conservatory background. Thus, effects may be different in other musicians, such as composers, singers, or rock/pop band members. Although other studies have reported that passively attending favored music induce analgesic effects on chronic pain patients (Garza-Villarreal et al., 2014), it has also been shown that professional musicians exhibit stress-like responses to music listening in contrast to non-musicians (Hassler, 2000); nevertheless in the current study we did not measure this effect and therefore cannot provide detailed information about music-induced analgesia function. Despite our efforts to accurately explain pain assessments, the ratio score method used to explore pain sensitiveness, combining pain thresholds and NRS pain ratings, may still depend on the participant’s comprehension of the task and reflect individual decisional factors about what has to be considered painful. Finally, this cross-sectional study does not allow conclusion about causal effects of music training and chronic pain on tactile thresholds and pain sensitivity but provides a framework for future longitudinal studies to assess this question.

In summary, our findings support the hypothesis that extensive sensorimotor training, such as playing an instrument in professional musicians, may lead to relevant changes in the processing of bodily information, which could be triggered either by increased peripheral somatosensory inputs or by central sensitization and loss of central endogenous pain control mechanisms as described in other chronic pain disorders.

## Author contributions

Anna M. Zamorano was involved in designing the study, recruitment of volunteers, acquisition of data and data analyses, and wrote the first draft of the manuscript. Inmaculada Riquelme was involved in designing the study, contributed to the preparation of the manuscript and critical revisions. Boris Kleber, Eckart Altenmüller and Samar M. Hatem were involved in preparing the manuscript and revising it for important intellectual content. Pedro Montoya was involved in designing the study, statistical data analyses and contributed to the preparation of the manuscript and critical revisions. All authors read and approved the final manuscript.

## Conflict of interest statement

The authors declare that the research was conducted in the absence of any commercial or financial relationships that could be construed as a potential conflict of interest.
